# Hydrogen Embrittlement Sensitivity of X70 Welded Pipe Under a High-Pressure Pure Hydrogen Environment

**DOI:** 10.3390/ma17235818

**Published:** 2024-11-27

**Authors:** Kangxin Shuai, Haixiao Liu, Ming Li, Shubiao Yin, Ba Li, Bing Wang, Qingyou Liu, Shujun Jia

**Affiliations:** 1 Faculty of Metallurgical and Energy Engineering, Kunming University of Science and Technology, Kunming 650031, China; 2 Engineering Steel Institute, Central Iron and Steel Research Institute, Beijing 100081, China; 3 SINOPEC Engineering Incorporation, Beijing 100101, China

**Keywords:** X70 pipeline steel, hydrogen embrittlement sensitivity, slow strain rate test, low cycle fatigue, microstructure

## Abstract

With the rapid development of hydrogen pipelines, their safety issues have become increasingly prominent. In order to evaluate the properties of pipeline materials under a high-pressure hydrogen environment, this study investigates the hydrogen embrittlement sensitivity of X70 welded pipe in a 10 MPa high-pressure hydrogen environment, using slow strain rate testing (SSRT) and low-cycle fatigue (LCF) analysis. The microstructure, slow tensile and fatigue fracture morphology of base metal (BM) and weld metal (WM) were characterized and analyzed by means of ultra-depth microscope, scanning electron microscope (SEM), electron backscattering diffraction (EBSD), and transmission electron microscope (TEM). Results indicate that while the high-pressure hydrogen environment has minimal impact on ultimate tensile strength (UTS) for both BM and WM, it significantly decreases reduction of area (RA) and elongation (EL), with RA reduction in WM exceeding that in BM. Under the nitrogen environment, the slow tensile fracture of X70 pipeline steel BM and WM is a typical ductile fracture, while under the high-pressure hydrogen environment, the unevenness of the slow tensile fracture increased, and a large number of microcracks appeared on the fracture surface and edges, with the fracture mode changing to ductile fracture + quasi-cleavage fracture. In addition, the high-pressure hydrogen environment reduces the fatigue life of the BM and WM of X70 pipeline steel, and the fatigue life of the WM decreases more than that of the BM as well. Compared to the nitrogen environment, the fatigue fracture specimens of BM and WM in the hydrogen environment showed quasi-cleavage fracture patterns, and the fracture area in the instantaneous fracture zone (IFZ) was significantly reduced. Compared with the BM of X70 pipeline steel, although the effective grain size of the WM is smaller, WM’s microstructure, with larger Martensite/austenite (M/A) constituents and MnS and Al-rich oxides, contributes to a heightened embrittlement sensitivity. In contrast, the second-phase precipitation of nanosized Nb, V, and Ti composite carbon-nitride in the BM acts as an effective irreversible hydrogen trap, which can significantly reduce the hydrogen embrittlement sensitivity.

## 1. Introduction

Against the background of “carbon peaking and carbon neutrality”, hydrogen energy has become a strategic choice to accelerate energy transformation, in which hydrogen transportation is a primary bottleneck limiting hydrogen energy applications. Realizing the low-carbon, sustainable development of the energy system, the use of pipeline transportation is an effective way to achieve large-scale, low-cost transportation [1,2,3,4]. However, pipeline steel exposed to pressurized hydrogen often suffers from reduced plasticity, toughness, and fatigue properties, impacting safety [5,6]. Especially welded joints, often the weakest points in pipeline systems, contain inclusions, pores, and cracks, increasing the risk of hydrogen damage. Therefore, hydrogen embrittlement sensitivity must be studied for high-pressure hydrogen-transporting pipeline steels.

Relevant studies have shown that the microstructure of pipeline steels can significantly affect their hydrogen embrittlement sensitivity, such as grain boundaries, dislocations, second-phase precipitation, and inclusions [7,8]. Depending on the varying binding energies between hydrogen atoms and different types of defects, these defects can be classified into two categories: reversible hydrogen traps and irreversible hydrogen traps. The hydrogen binding energy of reversible hydrogen traps is lower, and the trapped hydrogen atoms can easily escape then diffuse into the lattice of the metal, such as low-angle grain boundaries, dislocations, etc. [9,10]. Irreversible hydrogen traps have higher binding energies with hydrogen atoms, and it is difficult for hydrogen atoms to escape from the trap, such as inclusions, second-phase precipitation, etc. [11,12,13]. However, when assessing the hydrogen embrittlement sensitivity of pipeline steels, the number and distribution of hydrogen traps as well as their effects on hydrogen atom trapping, diffusion, and enrichment need to be considered comprehensively.

Currently, there are many research reports on the effect of hydrogen damage on pipeline steel under a hydrogen environment, but most of them are using electrochemical hydrogen charging [14,15]. Dong-Su Bae et al. [16] conducted research on the hydrogen embrittlement susceptibility of X70 pipeline steel by controlling the duration of hydrogen charging. The results indicated that as the hydrogen charging time increased the degree of hydrogen damage in X70 steel initially rose sharply and then leveled off. In addition, the current density of hydrogen charging also has a significant effect on the hydrogen embrittlement sensitivity of the steel: the higher the current density, the higher the hydrogen embrittlement sensitivity [14,17]. Although electrochemical hydrogen charging is able to simulate the hydrogen damage behavior of pipeline steel under specific conditions, hydrogen pipeline steel is faced with the effects of high-pressure gaseous hydrogen. The use of hydrogen filling in the gas phase is closer to the actual service conditions of hydrogen pipelines. In contrast, high-pressure hydrogen filling in the gas phase can couple the adsorption, dissociation, diffusion, and bias behavior of hydrogen infiltration into the specimen with the stress state of the pipeline steel, which is more directly responsive to the situation of the pipeline steel in the actual service operation environment.

The “ASME B31.12 Hydrogen Pipelines and Lines standard” recommends the use of low-grade pipes such as X42 and X52 for low and medium pressure conditions. However, in the future, large-scale, long-distance, and large-volume hydrogen transportation needs to use high-grade pipeline steels for high-pressure pure hydrogen transportation. When transporting hydrogen-containing media, the higher the strength of the pipe and the higher the hydrogen pressure, the more obvious the hydrogen damage phenomenon becomes [18,19]. Although there has been some progress in the related research on pure hydrogen environments, the hydrogen pressure is usually limited to low and medium pressures (6.3 MPa, 4 MPa) and below [20]. In contrast, few studies have been reported on pipeline steels in a high-pressure pure hydrogen environment at 10 MPa. Thorsten Michler et al. [21] showed that the hydrogen environment at 10 MPa resulted in a significant decrease in the hydrogen embrittlement sensitivity of X60 pipeline steels. Sina Rahimi et al. [19] investigated the effect of different hydrogen pressures on the hydrogen embrittlement sensitivity of X65 pipeline steels and pointed out that the hydrogen embrittlement sensitivity of X65 pipeline steels is significantly reduced when the hydrogen pressure reaches 10 MPa. D. Hejazi et al. [22] investigated the effect of manganese content on the hydrogen embrittlement sensitivity of X70 pipeline steel under 10 MPa high-pressure pure hydrogen environment by means of slow strain rate test, and the results showed that the hydrogen damage of X70 steel with higher manganese content was higher. Eun Ju Song et al. [23] studied the effect of tensile rate on the hydrogen embrittlement sensitivity of X70 pipeline steel in the 10 MPa pure hydrogen environment and pointed out that the hydrogen embrittlement sensitivity increased with the decrease of tensile rate. At present, there are no reports on the study of X70 pipeline steel base material and weld material under a 10 MPa high-pressure pure hydrogen environment, and the effect of its microstructure on the hydrogen damage is not clear.

In particular, most of the studies on high-pressure pure hydrogen transport pipeline steels have used laboratory small specimens, and there are few reports on the hydrogen embrittlement sensitivity of industrially piloted high-grade pipeline steels under a high-pressure pure hydrogen environment. Therefore, this paper investigates the hydrogen embrittlement sensitivity of base metal and weld metal from industrial production of X70 welded pipe under a 10 MPa high-pressure pure hydrogen environment by using hydrogen compatibility experiments such as slow strain rate test and low cycle fatigue, and it systematically explores the hydrogen damage mechanism of high-grade pipeline steels under a high-pressure pure hydrogen environment by combining the characterization methods such as 3D microscope, SEM, EBSD, and TEM.

## 2. Materials and Methods

The test material for a domestic steel pipe manufacturing company to provide X70 straight seam submerged arc welded pipe included pipe welding standards with reference to “GB/T 31032-2023 Welding and acceptance standard for steel pipings and pipelines”, specifications for Φ1016 mm × 17.5 mm, and chemical composition as shown in Table 1.

**Figure 1:**
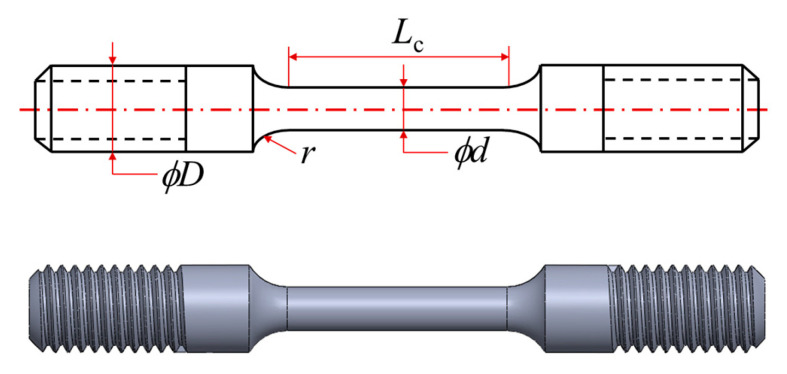
Schematic diagram of smooth tensile specimen (r = 3 mm; *ϕd* = 3 mm; *ϕD* = 6 mm; *L_c_* = 15 mm).

Slow strain rate testing (SSRT) was conducted on a WDML-3-30KN slow tensile testing machine, following “GB/T 39039-2020 Evaluation method for hydrogen-induced delayed fracture of high strength steels”. We set the BM and WM 2 types of specimens with the specimens parallel to the transverse direction of the pipe sampling and with specimen size shown in Figure 1. Specimens were cleaned with acetone and alcohol, then subjected to a strain rate of 2 × 10^−5^ s^−1^ in 10 MPa nitrogen and hydrogen environments.

**Figure 2:**
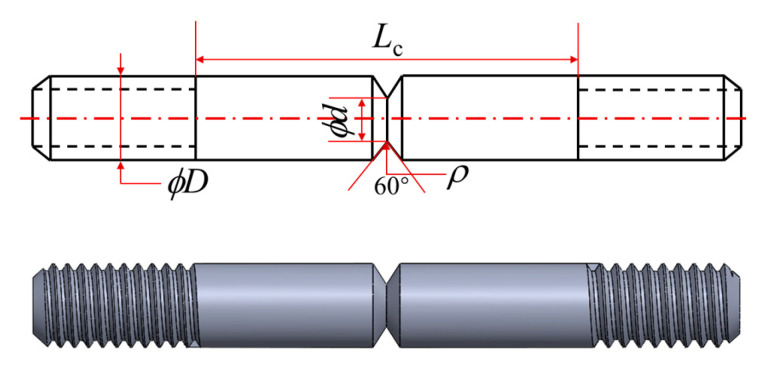
Schematic diagram of notched specimen (*ρ* = 0.25 mm; *ϕd* = 3 mm; *ϕD* = 6 mm; *L*_c_ = 25.4 mm).

The low cycle fatigue refers to “GB/T 34542.2-2018 Storage and transportation systems for gaseous hydrogen-Part 2: Test methods for evaluating metallic material compatibility in hydrogen atmosphere”, and notched specimens are used for the compatibility test with the hydrogen environment, which are all taken parallel to the transverse direction of the pipeline, and the dimensions of the specimens are as shown in Figure 2. Specimens were cleaned with acetone and alcohol to remove grease and debris, then tested in an autoclave under a 10 MPa nitrogen and hydrogen environment. A PLE-50 electric servo fatigue testing machine was used to carry out the low cycle fatigue. The load of the BM was 4043 N, the load of the WM was 4233 N, the stress ratio R = 0.1, and the loading waveform was a sinusoidal waveform with a frequency of 1 HZ.

A GP-300C (Shenzhen Simai Technology Co., Ltd, Shenzhen, CHN) stereo microscope was used to measure the area of slow tensile test fracture, and each measurement was taken several times and then averaged; a FEI Quanta 650FEG (Hillsboro, OR, USA) scanning electron microscope was used to observe the micro-morphology of the fracture; a VHX-7000N (KEYENCE, Osaka, JPN) ultra-depth microscope was used to observe the macro-morphology of the fracture on the front and the side; a Leica MEF-4M (Leica Microsystems, Wetzlar, GER) metallographic microscope, JSM-7900F (Oxford NordlysF+, Oxford, UK) thermal field emission scanning electron microscope, and Talos F200X G2 (Hillsboro, OR, USA) transmission electron microscope were used to finely characterize the microstructure of the BM and the WM. The formula for calculating the loss rate in nitrogen and hydrogen environments was as follows: Loss rate = (1−S_Hydrogen_/S_Nitrogen_) × 100%; (S is ultimate tensile strength (UTS), Reduction of area (RA), elongation (EL), number of failure cycles).

## 3. Results

### 3.1. Microstructure of X70 Pipeline Steel

**Figure 3:**
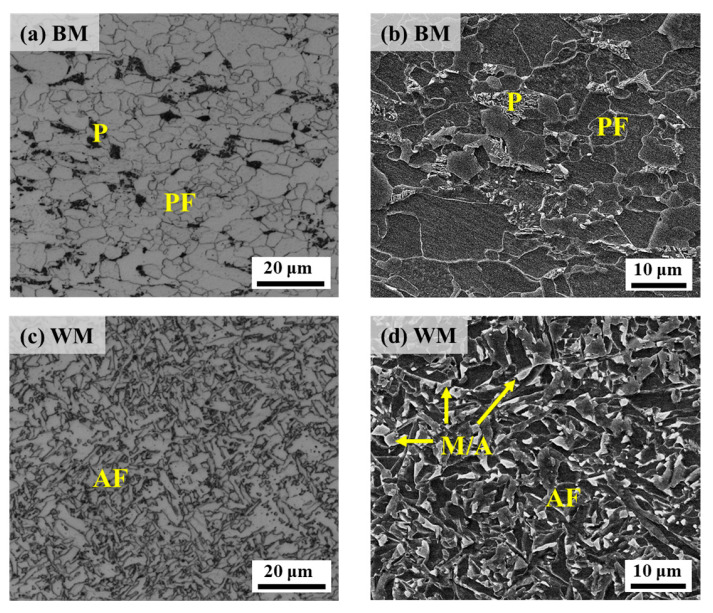
Microstructure of X70 welded pipe using OM (left column) and SEM (right column): (**a**,**b**) BM; (**c**,**d**) WM.

The microstructures of the BM and WM in X70 pipeline steel were analyzed and are shown in Figure 3, in which the BM (Figure 3a,b) consists mainly of polygonal ferrite (PF) + pearlite (P), and the WM (Figure 3c,d) is predominantly acicular ferrite (AF), with a large number of Martensite/austenite (M/A) constituents seen.

### 3.2. Slow Strain Rate Test Result

**Figure 4:**
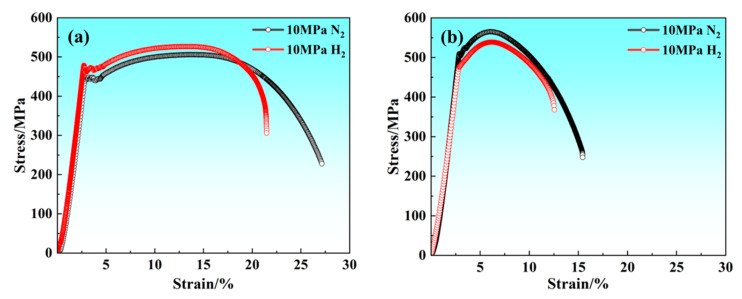
Stress-strain curves of BM and WM: (**a**) BM; (**b**) WM.

Figure 4a,b show the slow strain rate testing stress-strain curves of X70 pipeline steel BM and WM, respectively. Compared to 10 MPa nitrogen, the UTS of BM in 10 MPa hydrogen shows a minor increase, while EL decreases significantly; the UTS and EL of the WM in the 10 MPa hydrogen environment are lower than that in the 10 MPa nitrogen environment.

**Figure 5:**
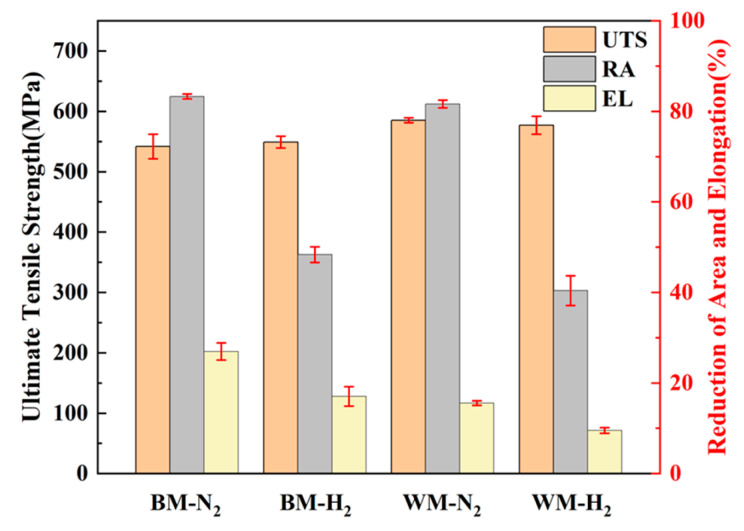
Slow strain rate test results of BM and WM in nitrogen and hydrogen environments.

The results of the slow tensile comparison of BM and WM under nitrogen and hydrogen environments are given in Figure 5. It can be seen that the UTS of BM and WM under nitrogen and hydrogen environments do not change greatly. The results of related research show that the gas environment does not have a significant effect on the strength of specimens. Among them, the RA and EL of BM and WM in the hydrogen environment are much lower than that in the nitrogen environment as a whole, and the comparison can be seen that the loss rate of UTS and EL of BM in a high-pressure hydrogen environment is not much different from that of WM, whereas the degree of RA of WM is very significant. The specific slow strain rate testing data are given in Table 2, and the loss rates of UTS, RA, and EL of the BM specimens are −1.3%, 41.9%, and 37.0%, respectively; the loss rates of UTS, RA, and EL of the WM specimens are 1.4%, 50.5%, and 39.1%, respectively.

**Figure 6:**
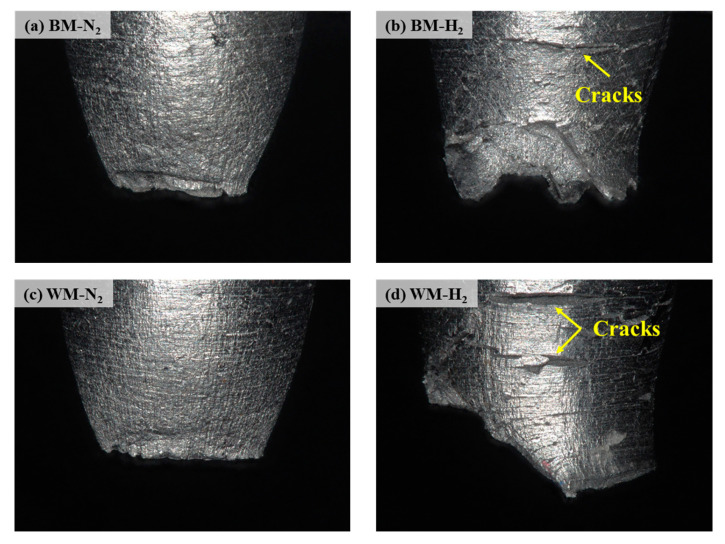
Macroscopic morphology of the fracture side of BM and WM: (**a**) BM-N_2_; (**b**) BM-H_2_; (**c**) WM-N_2_; (**d**) WM-H_2_.

**Figure 7:**
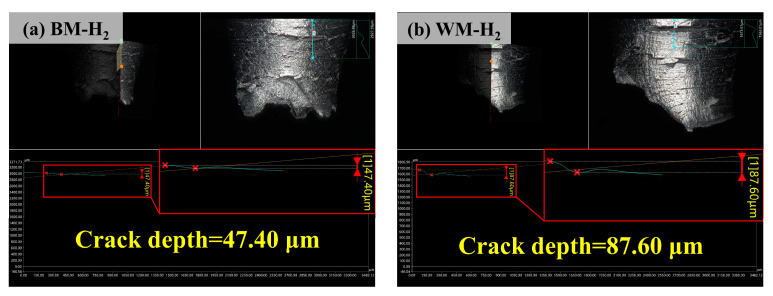
Crack depths of BM and WM in 10 MPa hydrogen environment: (**a**) BM; (**b**) WM.

The macroscopic morphology of the fracture side of the BM and WM under 10 MPa nitrogen and 10 MPa hydrogen was observed by using an ultra-depth microscope. As shown in Figure 6, the BM and WM showed clear necking, with fracture surfaces that were relatively flat under nitrogen conditions (Figure 6a,c); the fracture surfaces of the BM and WM were uneven and jagged under hydrogen (Figure 6b,d), and obvious annular cracks were observed on the fracture sides, which were typical hydrogen induced cracks. The maximum value of the crack depth was taken after measuring the depth of the annular crack, as shown in Figure 7, the maximum crack depth of the BM specimen was 47.40 μm, and the maximum crack depth of the WM specimen was 87.60 μm.

**Figure 8:**
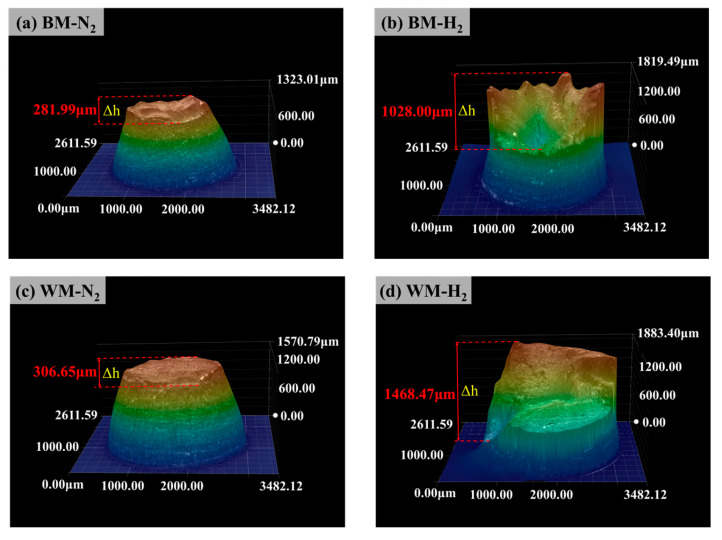
3D diagrams of the fracture of the BM and WM: (**a**) BM-N_2_; (**b**) BM-H_2_; (**c**) WM-N_2_; (**d**) WM-H_2_.

Figure 8 gives the 3D diagrams of the fracture (fracture’s color from highest to lowest: orange, green, blue) of BM and WM specimens under 10 MPa nitrogen and 10 MPa hydrogen environments. Overall, it can be seen that the hydrogen environment increases the fracture surface unevenness significantly compared to the nitrogen environment. Measurement of the fracture unevenness (Δh) of the specimens revealed that compared with the nitrogen environment (281.99 μm), the fracture unevenness of the BM specimens in the hydrogen environment increased significantly (1028.00 μm); the fracture unevenness of the WM specimen in the nitrogen environment was 306.65 μm, while the fracture unevenness in the hydrogen environment reached 1468.47 μm. Notably, fracture surface unevenness for WM specimens in both nitrogen and hydrogen environments is higher than for BM specimens.

**Figure 9:**
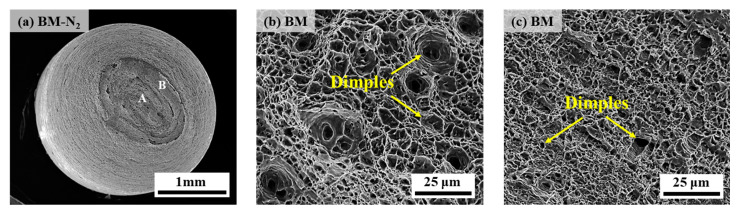
SEM diagrams of the fracture of the BM specimen in 10 MPa nitrogen: (**a**) overall low-power morphology; (**b**) position A; (**c**) position B.

**Figure 10:**
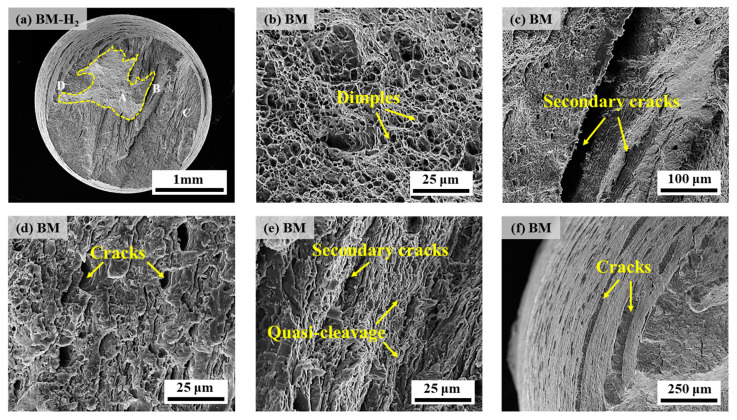
SEM diagrams of the fracture of the BM specimen in 10 MPa hydrogen: (**a**) overall low-power morphology; (**b**) position A; (**c**,**d**) position B; (**e**) position C; (**f**) position D.

Figure 9 and Figure 10 show the fracture scanning morphology of the X70 pipeline steel BM specimen under 10 MPa nitrogen and 10 MPa hydrogen environments, respectively. Observation of Figure 9a shows that the fracture surface of the X70 pipeline steel BM is characterized by an elliptical cup and cone shape, indicating that significant necking occurred before fracture, which includes a concave and convex smooth region at the heart and a shear lip with a 45° edge. Dimple morphology varied between the center (Figure 9b) and edge regions (Figure 9c), with equiaxed dimples at the center and elongated dimples at the edge. Therefore, the X70 pipeline steel of the BM specimen shows good plasticity in 10 MPa nitrogen, which is typical of ductile fracture.

Figure 10 shows the scanned fracture morphology under a 10 MPa hydrogen environment. From Figure 10a, it can be seen that the fracture surface is relatively flat on the whole, showing slight necking and obvious dimple morphology can be observed in the dashed box (Figure 10b). More secondary cracks can be observed near the center area (Figure 10c), and the enlargement of the area around the cracks (Figure 10d) shows that the overall fracture morphology is relatively chaotic, the dimple morphology has basically disappeared, and only small micropores can be observed in local areas, but they also do not have the typical characteristics of the cleavage fracture. Close observation reveals that the area around the tiny micropores is very flat, accompanied by a large number of microcracks, indicating that the contribution to plastic deformation during fracture is relatively small. Hydrogen exposure resulted in quasi-cleavage characteristics, replacing the ductile dimple morphology found in nitrogen conditions. Further observation of the fracture edge location (Figure 10e) shows numerous microcracks and exhibits obvious quasi-cleavage fracture characteristics. At the fracture side (Figure 10f), more annular cracks perpendicular to the tensile direction can be observed, in which the annular cracks may arise due to the hydrogen atoms attaching here and being trapped by hydrogen traps in the metal such as dislocations, inclusions, grain boundaries, etc., resulting in localized stress concentration at this location. On the whole, most of the areas show the characteristics of quasi-cleavage fracture except the dimple morphology near the fracture heart part. Therefore, the fracture form of the X70 pipeline steel BM specimen in 10 MPa hydrogen is quasi-cleavage fracture + ductile fracture.

**Figure 11:**
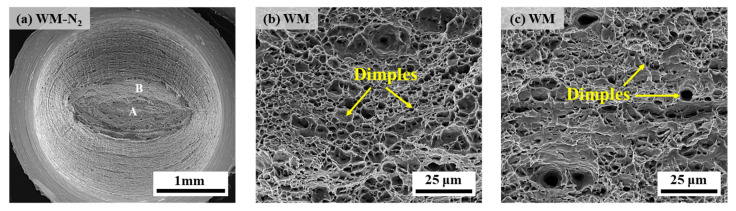
SEM diagrams of the fracture of the WM specimen in 10 MPa nitrogen: (**a**) overall low-power morphology; (**b**) position A; (**c**) position B.

**Figure 12:**
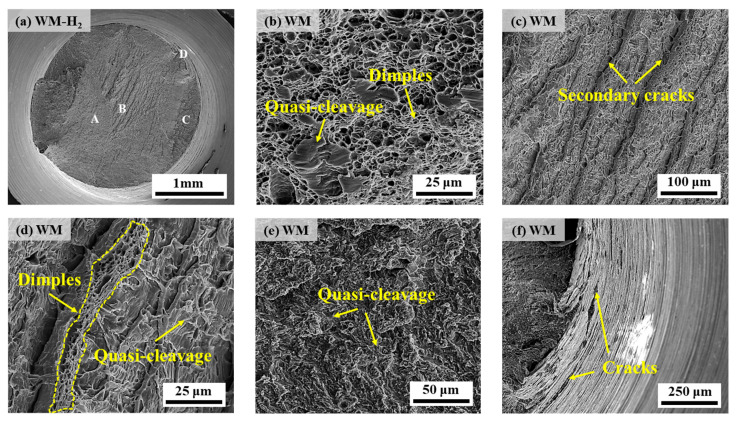
SEM diagrams of the fracture of the WM specimen in 10 MPa hydrogen: (**a**) overall low-power morphology; (**b**) position A; (**c**,**d**) position B; (**e**) position C; (**f**) position D.

Figure 11 and Figure 12 show the scanned fracture morphology of the X70 pipeline steel WM specimen in 10 MPa nitrogen and 10 MPa hydrogen, respectively. As can be seen from Figure 11a, the fracture morphology of the WM specimen under nitrogen is similar to that of the BM, which is a typical cup and cone shape, and no radial zone is observed, indicating that its plasticity is as well. Enlargement of the WM fracture (Figure 11b,c) shows the center of the smooth region for the size of the depth of the equiaxial dimple morphology and the shear lip for the dimple morphology with a certain similarity to the shape of the parabola. Therefore, X70 pipeline steel WM in the 10 MPa nitrogen fracture has characteristics of the typical ductile fracture.

The scanning diagrams under a hydrogen environment are given in Figure 12. The fracture shows slight necking, and the shear lip on the fracture surface is not obvious, but the dimple morphology can still be observed (Figure 12b), in which the dimple size and number are small and few compared with that under a nitrogen environment. Many shallow secondary cracks can be seen near the fracture heart (Figure 12c), and the cracks can be seen clearly when zoomed in (Figure 12d). Magnifying the crack, the quasi-cleavage morphology can be seen, which is the distinct steps in the fracture surface due to rapid tearing, and a row of dimple morphology can be seen near the cracks (shown in the dashed box of Figure 12d); the quasi-cleavage morphology near the edge of the fracture (Figure 12e) is even more obvious; and the annular cracks can be observed on the surface of the specimen (Figure 12f). Therefore, except for the dimple morphology near the center, the WM as a whole showed quasi-cleavage fracture characteristics under a 10 MPa hydrogen environment and exhibited the fracture mode of quasi-cleavage fracture + ductile fracture.

### 3.3. Low Cycle Fatigue Result

**Figure 13:**
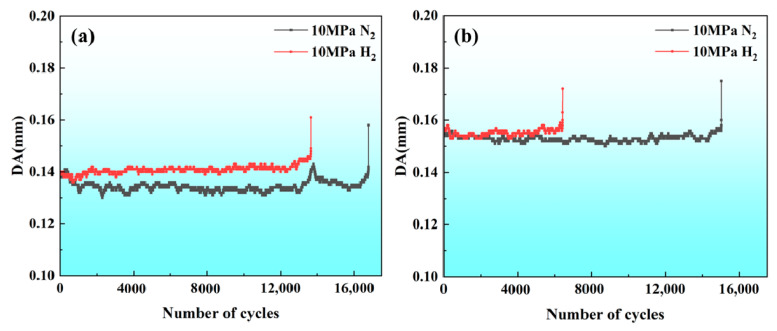
Relationship between the number of failure cycles and DA for X70 pipeline steel: (**a**) BM; (**b**) WM.

Displacement amplitude (DA) is the difference between the displacement obtained at the maximum stress and the minimum stress during a cycle of loading. The DA value remains stable, which means that the crack in the specimen is at the stage of germination; the point where the DA value starts to rise means that the crack is starting to germinate; and the DA value continuing to grow means that the crack is expanding [24]. Figure 13 shows the relationship between the number of failure cycles and DA for X70 pipeline steel BM and WM specimens in 10 MPa nitrogen and 10 MPa hydrogen environments. In the nitrogen environment (Figure 13a), the DA value of the BM specimen starts to rise after 1100 cycles, which is the point of crack initiation, and then it drops and then rises again. The DA value remains stable after that, and then it starts to rise again after 13,000 cycles, but starts to drop after 14,000 cycles until the specimen fracture occurs after 16,795 cycles, which suggests that the BM specimen under a nitrogen environment starts to crack at the early stage of the LCF, but it does not expand into a large crack. The DA value starts to decrease after the formation of a small crack, which consumes part of the energy. Under a hydrogen environment (Figure 13a), the point of crack initiation can be observed after 700 cycles, i.e., the hydrogen environment accelerates the formation of cracks, and the cracks have been in the initiation gestation period, while the DA value continues to increase until the specimen breaks after 12,000 cycles.

Under the nitrogen environment (Figure 13b), the DA value of the WM specimen starts to increase and then decrease after 3000 cycles, more micro-cracks are generated between 3000 and 14,000 cycles, and the DA value continues to increase until the specimen fractures after 14,000 cycles. In a hydrogen environment (Figure 13b), the point of crack initiation can be observed in the WM specimen after 1000 cycles, but the micro-crack formation did not continue to expand, and after 6000 cycles, the DA value increased continuously. Thus, the fatigue crack stopping ability of the specimen decreased. By comparing the number of fatigue life cycles of BM and WM specimens under 10 MPa nitrogen and 10 MPa hydrogen environments, it can be seen (Table 3) that the number of failure cycles of the BM specimen under a hydrogen environment is 13,654 cycles, compared with a nitrogen environment (16,795 cycles), with the loss rate of 18.70%; for the WM specimen in the hydrogen environment it is 6437 cycles, compared with the loss rate of 57.36% in nitrogen environment (15,096 cycles).

**Figure 14:**
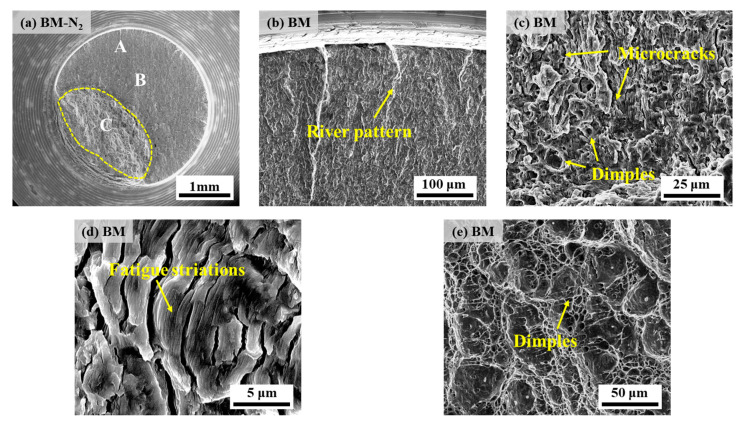
Fatigue fracture morphology of BM specimen in 10 MPa nitrogen: (**a**) overall low-power morphology; (**b**) position A; (**c**,**d**) position B; (**e**) position C.

**Figure 15:**
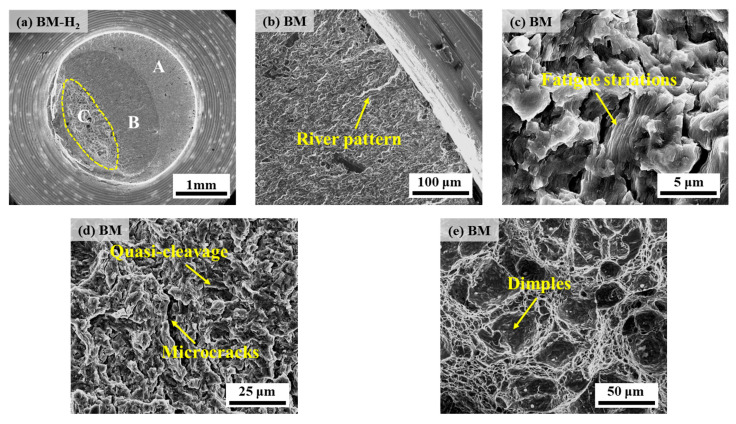
Fatigue fracture morphology of BM specimen in 10 MPa hydrogen: (**a**) overall low-power morphology; (**b**,**c**) position A; (**d**) position B; (**e**) position C.

Figure 14 and Figure 15 show the fatigue fracture morphology of X70 pipeline steel BM specimens in 10 MPa nitrogen and 10 MPa hydrogen environments, respectively. Each fracture consists of the fatigue crack initiation zone (FCIZ), fatigue crack propagation zone (FCPZ), and instantaneous fracture zone (IFZ), with IFZ surfaces displaying dimple morphology in both nitrogen and hydrogen environments [24]. In the FCIZ, both nitrogen and hydrogen environments showed typical river-like patterns (Figure 14b and Figure 15b), which reflected the initial characteristics of crack initiation. Upon entering the FCPZ, obvious microcracks can be seen (Figure 14c and Figure 15d), and the formation of these microcracks consumes a large amount of energy, thus inhibiting the crack extension to some extent. However, in the hydrogen environment, the crack extension speed is obviously accelerated, and an obvious demarcation line can be seen on the fracture surface when the crack extension reaches the threshold value. In addition, the interior of the specimens under the hydrogen environment also showed obvious quasi-cleavage morphology characteristics (Figure 15d). It is especially noteworthy that in the nitrogen environment, obvious fatigue striations can be observed in region B for the BM specimen (Figure 14d), while in the hydrogen environment, fatigue striations (Figure 15c) can be observed in region A, which further indicates that the hydrogen environment accelerates the crack formation. Specifically, the crack initiation point of the specimen under the hydrogen environment can be observed after reaching 700 fatigue cycles. Finally, in the IFZ, obvious dimple morphology can be observed for specimens in both environments (Figure 14e and Figure 15e). However, the IFZ area of the specimens in the hydrogen environment is smaller than in the nitrogen environment, which indicates that the specimens fracture earlier in the hydrogen environment, i.e., the hydrogen environment reduces their fatigue life.

**Figure 16:**
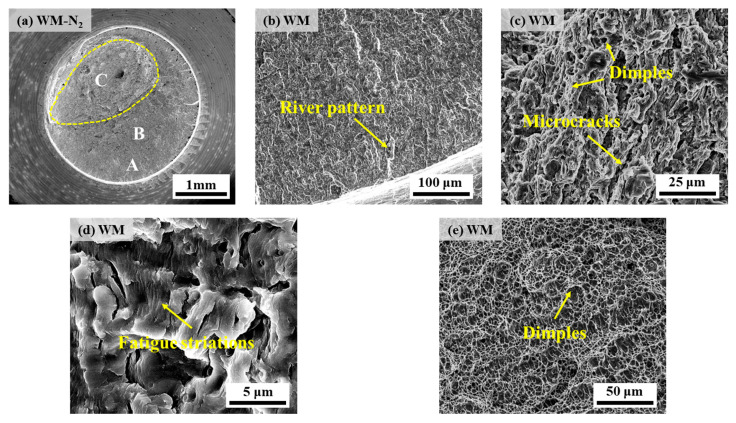
Fatigue fracture morphology of WM specimen in 10 MPa nitrogen: (**a**) overall low-power morphology; (**b**) position A; (**c**,**d**) position B; (**e**) position C.

**Figure 17:**
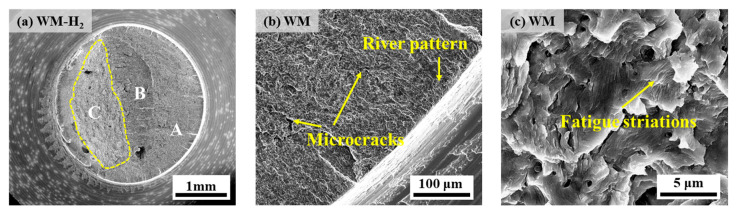

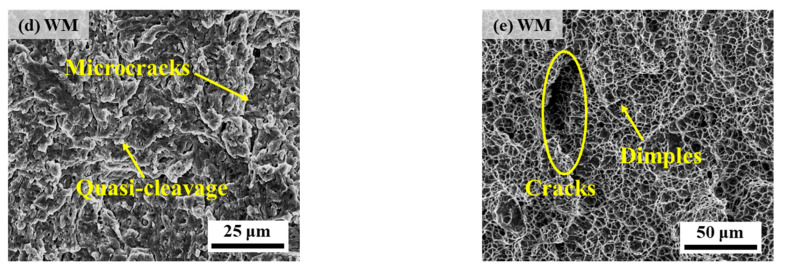
Fatigue fracture morphology of WM specimen in 10 MPa hydrogen: (**a**) overall low-power morphology; (**b**,**c**) position A; (**d**) position B; (**e**) position C.

Figure 16 and Figure 17 show the fatigue fracture morphology of X70 pipeline steel WM specimens in 10 MPa nitrogen and 10 MPa hydrogen, respectively. The fatigue fracture morphology is similar to that of BM, and both show obvious river patterns in the FCIZ (Figure 16b and Figure 17b). Moreover, some fine microcracks and fatigue striations can be seen in the WM specimen at the early stage of the test under a hydrogen environment (Figure 17c), which indicates that the crack formation and extension are particularly rapid under a hydrogen environment. In the FCPZ, due to the continuous action of cyclic loading, a large number of irregular microcracks appeared on the fracture surface (Figure 16c and Figure 17d), which led to the stress concentration in the region and further accelerated the crack extension. Especially notably, the dimple morphology of the WM specimen basically disappeared under the hydrogen environment, showing certain quasi-cleavage fracture morphology characteristics. Finally, in the IFZ, the area of the WM fracture in the hydrogen environment is significantly reduced compared with that in the nitrogen environment, which intuitively reflects that the hydrogen environment substantially shortens the number of fatigue life cycles.

## 4. Discussion

Due to the unique metallurgical properties of welds, hydrogen atoms tend to accumulate, leading to potential pipeline cracking. At present, scholars at home and abroad have carried out some experimental studies on the compatibility of pipeline welds under the hydrogen environment. Some studies have shown that in high-strength pipeline steel the hydrogen trap density in the weld zone is significantly higher than the base material [25]. Lee et al. [26] addressed the “local embrittlement phenomenon” in the pipeline weld. Using the electrochemical hydrogen charging method of X70 pipeline steel research, the results show that the impact toughness of the weld zone after hydrogen charging is greatly reduced. In this article, a series of research results from Figure 4, Figure 5, Figure 6, Figure 7, Figure 8, Figure 9, Figure 10, Figure 11, Figure 12, Figure 13, Figure 14, Figure 15, Figure 16 and Figure 17 also show that the hydrogen embrittlement sensitivity of the WM in the X70 welded pipe is higher than that of the BM. It is well known that M/A constituents, inclusions, and dislocations are strong hydrogen trapping points in steel [27,28,29], which are prone to exacerbate the hydrogen damage behavior of pipelines, and the difference in the binding energy between hydrogen traps of different sizes and morphologies and hydrogen is relatively obvious. In order to improve the hydrogen damage resistance of pipeline steel, the effect of microstructure on hydrogen embrittlement sensitivity should be fully considered. Therefore, this study discusses and analyzes the hydrogen embrittlement sensitivity of BM and WM from the microstructure aspect concerning the results of hydrogen compatibility tests in X70 welded pipes.

### 4.1. Effect of Grain Size

**Figure 18:**
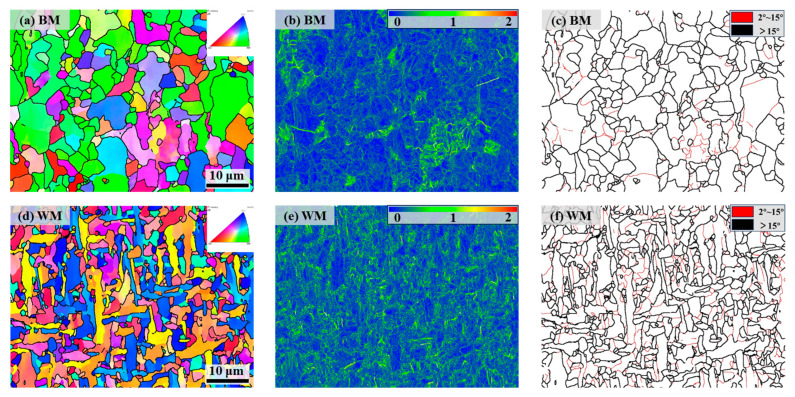
EBSD results of BM (**a**–**c**) and WM (**d**–**f**) specimens: (**a**,**d**) IPF diagrams; (**b**,**e**) KAM diagrams; (**c**,**f**) high and low angle grain boundaries diagrams.

**Figure 19:**
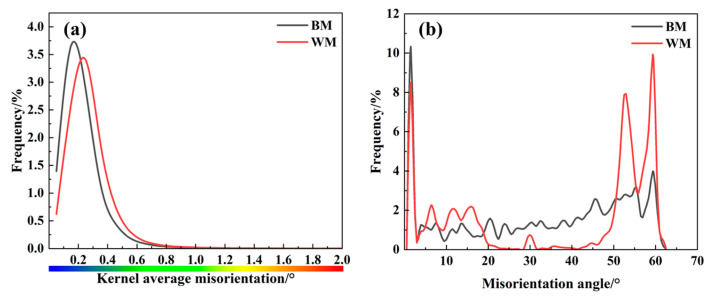
Statistical results of EBSD for BM and WM specimens: (**a**) KAM distribution curves; (**b**) grain boundary misorientation distribution.

The EBSD diagram in Figure 18 shows that the average grain size of the BM is about 3.02 μm, while the average grain size of the WM is about 1.92 μm, which is finer than that of the BM. The BM’s microstructure shows irregular grain sizes, while the WM’s structure is more uniform. Related research results show that hydrogen diffusion in the material is closely related to the grain size: the finer and more uniform the grain size, the better its resistance to hydrogen embrittlement [30]. Yazdipour et al. [31] used a two-dimensional (2D) model to study the effect of grain size on the diffusion of hydrogen in X70 pipeline steel, and the results show that when the grain size is less than 46 μm the rate of hydrogen diffusion with the reduction of the grain size decreases. The KAM diagram (Figure 18b,e and Figure 19a) shows that the local geometric dislocation densities of the BM and the WM do not differ much, but the dislocation density distribution of the WM is more uniform. Dislocations as reversible hydrogen traps and hydrogen diffusion channels tend to reduce the hydrogen embrittlement sensitivity of steel [32]. It has been shown that for hydrogen-filled X70 pipeline steel, in the region of coarse ferrite grain and low dislocation density, hydrogen is more likely to accumulate in the stress concentration zone near the crack tip, which accelerates the cracking in this region [33]. Statistics on the grain boundary misorientation between the BM and the WM (Figure 19b) show that although the WM contains a high number of high-angle grain boundaries, which can be enriched with a certain amount of hydrogen, it does not lead to an increase in the local hydrogen concentration due to its uniform distribution. Comparatively, low-angle grain boundaries act as reversible hydrogen traps [32,34], which have an important effect on the hydrogen embrittlement sensitivity of steel. Due to the low hydrogen capture energy, low grain boundary energy, and high hydrogen trap density of low-angle grain boundaries, hydrogen atoms are easily trapped even at very low hydrogen concentrations to reach the critical concentration for hydrogen embrittlement. However, the captured hydrogen atoms can easily get out of the hydrogen trap and enter the lattice; at the same time, low-angle grain boundaries act as channels, allowing hydrogen diffusion and affecting embrittlement sensitivity. Hydrogen atoms only need to overcome the lower energy barrier for further diffusion and aggregation, thus affecting the material’s hydrogen embrittlement sensitivity. Therefore, although dislocations and low-angle grain boundaries as reversible hydrogen traps in the microstructure of BM and WM increase their hydrogen damage, the percentage of dislocations and low-angle grain boundaries in both of them does not differ much, compared to which WM with smaller grain sizes improve their hydrogen embrittlement resistance to a certain extent.

### 4.2. Effect of M/A Constituents

**Figure 20:**
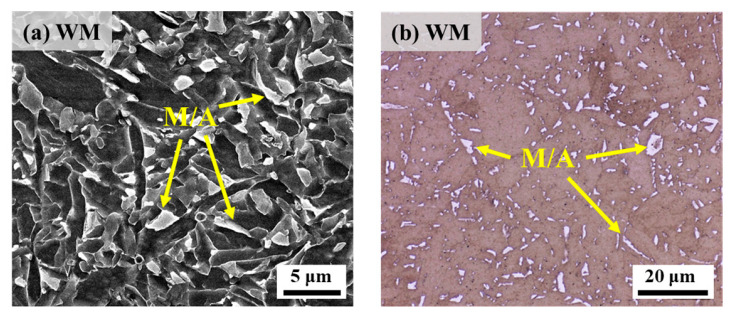
Microstructure of M/A constituents in the WM: (**a**) high-power SEM diagram of M/A constituents; (**b**) OM diagram of the M/A constituents corroded by Lepera reagent.

M/A constituents are usually harder than ferrite and bainite matrix microstructure due to the effect of containing martensitic microstructure, and this difference in hardness can lead to uneven stresses in the specimen during the tensile process and concentration of stresses near the M/A constituents, which can lead to cracking [35,36]. The microstructure in Figure 20a,b shows that the WM contains a large number of large-sized M/A constituents. Among them, the M/A constituents, as hard-phase microstructure, are prone to cause uneven soft and hard phases in the tensile specimen, which lead to the formation of secondary cracks on the fracture surface of the specimen. The results of related studies show that with M/A constituents as strong hydrogen trapping sites [37,38], hydrogen atoms are easily enriched, and when the hydrogen concentration reaches a critical value, M/A constituents may be the preferred site for HE formation. Zhang et al. [29] used the SSRT test and in-situ electrochemical hydrogen charging method to study the hydrogen embrittlement sensitivity of X65 pipeline steel and found that the M/A constituents are the most important factor influencing the high susceptibility to hydrogen embrittlement of acicular ferritic-based WM with high hydrogen embrittlement sensitivity. Therefore, according to the results of SSRT and LCF experiments in this paper, it can be seen that a large number of M/A constituents may be an important factor contributing to the high hydrogen embrittlement sensitivity of the WM specimen.

### 4.3. Effect of Inclusions

**Figure 21:**
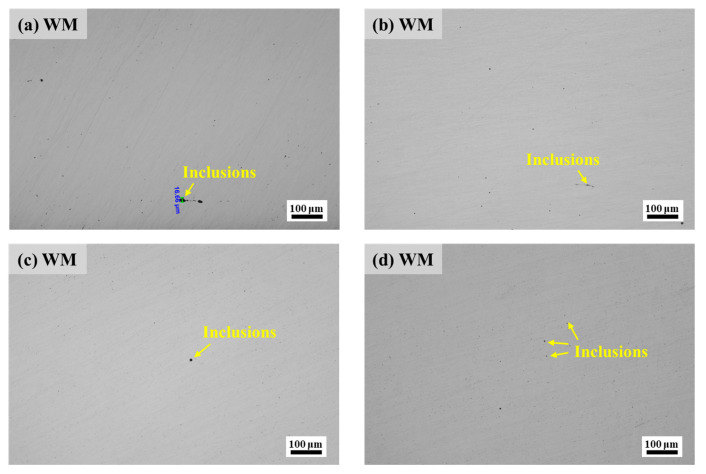
Results of steel inclusions rating for WM specimen: (**a**) B extra wide; (**b**) B fine grade 0.5; (**c**) D coarse grade 0.5; (**d**) D fine grade 1.0.

**Figure 22:**
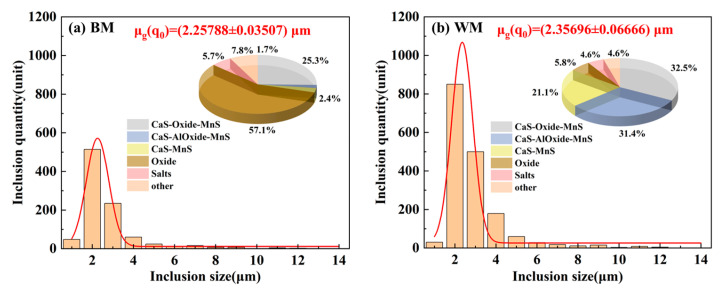
Distribution of number, size, and type of inclusions in BM and WM specimen steels: (**a**) BM; (**b**) WM.

Inclusion rating on the steel surface of the WM specimen was carried out, and several fields of view were selected for observation, as shown in Figure 21. From the figure, it can be seen that the WM specimen surface was mainly detected as having Class B (alumina-like) inclusions and Class D (spherical oxide-like) inclusions, in which the width of alumina-like inclusions reached 16.66 μm (Figure 21a). To further determine the size and type of inclusions in the BM and WM specimen steels, an Aspex SEM was used to scan the non-metallic inclusions on an area of 92.160 mm^2^ of the test steel. Among them, a total of 613 non-metallic inclusions were detected in the BM sample steel (Figure 22a), with the inclusions mainly distributed in the 2–3 μm range, and the inclusions were mainly Oxide inclusions and CaS-Oxide-MnS inclusions, with a proportion of 57.0% and 25.3%, respectively; 1463 non-metallic inclusions were detected in the WM sample steel (Figure 22b), which is about 2.4 times larger than that of the BM. The size of inclusions was mainly distributed in the range of 2~4 μm, and the types of inclusions were CaS-Oxide-MnS inclusions, CaS-AlOxide-MnS inclusions, and CaS-MnS inclusions, the proportions of which were 32.5%, 31.4% and 21.1%, respectively.

The results of related studies have shown that inclusions, as irreversible hydrogen traps, have an important effect on the hydrogen embrittlement sensitivity of steel [39,40,41]. For example, MnS inclusions usually lead to hydrogen-induced crack initiation in steel [42,43], and the presence of some Al-rich inclusions in steel also has an important effect on hydrogen embrittlement sensitivity [40]. It has been pointed out in the literature that hydrogen atoms diffused into the steel will be captured by the inclusions or the interface between the inclusions and the substrate, and the aggregation of hydrogen atoms at the site will lead to the formation of hydrogen pressure. When the hydrogen pressure is more than the critical hydrogen pressure at the site, then it will result in the crack initiation, and with the further increase of applied stresses, more hydrogen will be aggregated around the crack tip, which will then promote the crack extension [44,45]. Moreover, inclusions with a size larger than 2 μm will increase the hydrogen crack nucleation [46]. In this article, a large number of inclusions such as large-sized MnS and oxides of Al were also detected in the BM and WM specimen steels, which act as hydrogen trapping points that can easily cause crack initiation, and under the action of tensile and cyclic loading stresses, hydrogen atoms are enriched at the cracks, resulting in the further extension of the cracks, i.e., the higher number of tensile and fatigue fracture specimens were observed in Figure 10, Figure 12, Figure 15, and Figure 17, were of secondary cracks. Therefore, the presence of inclusions in the steel has an adverse impact on the BM and WM specimen steel resistance to hydrogen embrittlement. By analyzing the type and number of inclusions it can be seen that the WM at the MnS inclusions and Al-rich oxides inclusions are more than the BM, which is also an important reason for the hydrogen damage of the WM. 

### 4.4. Effect of Second-Phase Precipitation

**Figure 23:**
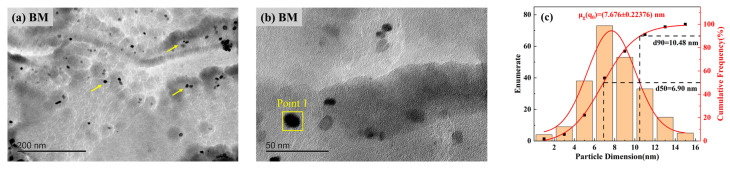

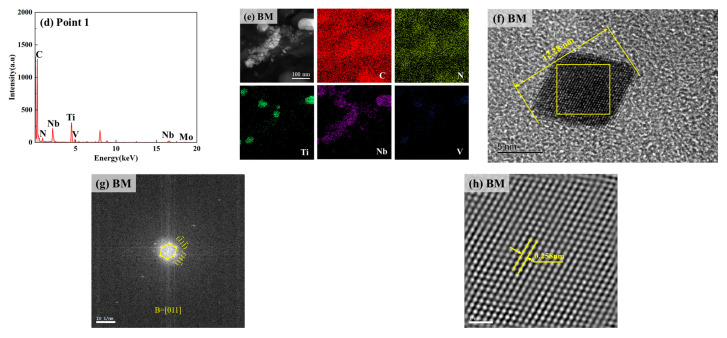
TEM characterization of steel extraction replica and EDS analysis in BM specimen: (**a**) low magnification of precipitated phase morphology; (**b**) high magnification of precipitated phase morphology; (**c**) particle size distribution of the precipitated phase; (**d**) point sweep of the EDS energy spectrum; (**e**) surface sweep of the EDS energy spectrum; (**f**) bright-field diagram; (**g**) FFT; (**h**) IFFT.

**Figure 24:**
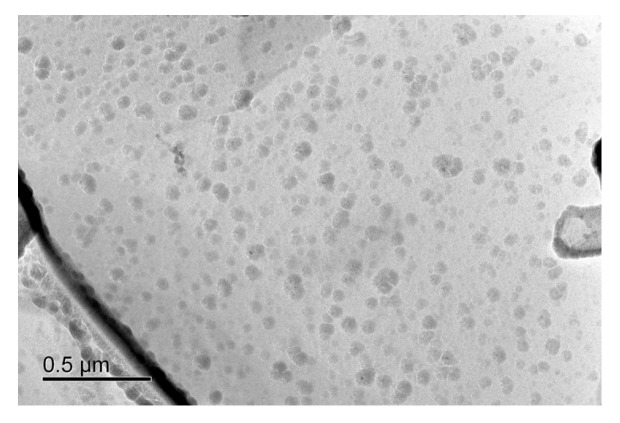
TEM characterization of steel extraction replica in WM specimen.

The second-phase precipitation behavior of the BM and WM specimen steels was investigated using the extraction replica method, as shown in Figure 23 and Figure 24. From Figure 23a, it can be seen that the precipitated phases of the BM specimen steel are diffusely distributed and most of the sizes are distributed in the 4–12 nm interval (Figure 23c), and the EDS energy spectra show (Figure 23d,e) that the types of precipitated phases are mainly the composite precipitation of (Ti, Nb, V)x(C, N)y. The high-resolution atomic diagram of the precipitated phase was characterized by high-resolution transmission electron microscopy (HRTEM) (Figure 23f), and the size of the precipitated phase was about 12.28 nm, with the Fourier Transform (FFT) and the Inverse Fourier Transform (IFFT) using the Digital Micrograph 3.5 software, which shows that the second-phase precipitation is mainly a face-centered cubic structure, and it was determined that the [011] interplanar spacing of the crystal face is around 0.258 nm. The TEM characterization of the WM specimen steel is shown in Figure 24, in which no nanoscale second-phase precipitation was detected.

Haq et al. [47] found that high-density precipitation of Ti, Nb carbon, and nitrogen compounds can reduce the hydrogen diffusion coefficient of X70 pipeline steel. Mohtadi-Bonab et al. [48] also demonstrated that carbons and nitrides of M(C, N) (M: Nb or Ti) smaller than 100 nm can pin the dislocations and reduce their mobility, thus increasing the resistance to hydrogen embrittlement sensitivity. It has also been shown that the second-phase precipitation of vanadium-containing nanoscale carbon-nitrides acts as irreversible hydrogen traps with high binding energies to capture diffusible hydrogen atoms [49,50,51]. Therefore, it can be seen from Figure 23 that the carbon and nitrogen in the BM specimen steel will form a carbonitride composite second-phase precipitation with elements such as niobium, vanadium, and titanium, and these fine nanoscale carbonitride particles can act as effective hydrogen traps to bind hydrogen, impede the diffusion and migration of hydrogen, and lead to a more uniform distribution of hydrogen to reduce the hydrogen content in the lattice, thus enhancing the hydrogen embrittlement sensitivity. It is known from the HELP mechanism [25,52] that hydrogen can promote the emission and movement of dislocations, and when the hydrogen concentration is higher than the critical value, then hydrogen-induced plastic deformation is induced, and hydrogen-induced cracks are formed at the dislocation buildup. Combined with the fracture morphology in Figure 10 and Figure 12, some tear ridge morphology can be seen in the QC area of the BM and WM, which indicates hydrogen-enhanced local plasticity (HELP). The HEDE mechanism suggests [25,52] that localized aggregation of hydrogen atoms reduces the bonding force between metal atoms, which can easily lead to hydrogen-induced fractures under the action of applied stresses. From the test results of SSRT and LCF, it can be seen that the hydrogen embrittlement sensitivity of the BM is lower than that of the WM, and one of the important reasons may be that the HEDE mechanism is suppressed in the BM, and a large number of precipitated phases of Nb, V, and Ti carbon-nitrogen compounds act as effective hydrogen traps, which homogenize the distribution of hydrogen and reduce the hydrogen embrittlement property of the BM. Therefore, the formation of precipitated phases increased the resistance to hydrogen embrittlement sensitivity of the BM material specimen steel.

## 5. Conclusions

In this article, the tensile and fatigue properties of X70 welded pipes under 10 MPa nitrogen and 10 MPa hydrogen environments were investigated by using the method of hydrogen filling in the gas phase and combined with the characterization of OM, SEM, EBSD, and TME to reveal the connectivity between microstructure and hydrogen embrittlement sensitivity to draw the following conclusions:

1. SSRT test results show that after hydrogen filling in the gas phase, in 10 MPa hydrogen, the RA and EL of both BM and WM decreased, with WM showing a significantly greater RA reduction. The tensile fracture shows that the fractures of BM and WM specimens under the nitrogen environment are relatively flat, and the fracture mode is ductile fracture, while the fracture unevenness of the BM and WM specimens under the high-pressure hydrogen environment increases, and a large number of microcracks appear on the fracture surface and fracture edges, with the fracture mode changing to ductile fracture + quasi-cleavage fracture.
2. LCF test results show that after hydrogen filling in the gas phase, the fatigue life cycles of BM dropped by 18.7%, while WM cycles dropped by 57.4%, indicating greater fatigue degradation in WM. The fatigue fracture shows that compared with nitrogen, the X70 pipeline steel BM and WM specimens show quasi-cleavage fracture morphology in the hydrogen environment, and the fracture dimpled area in the IFZ is greatly reduced.
3. The difference in hydrogen embrittlement sensitivity between the BM and WM of X70 pipeline steel is mainly reflected in the microstructure. Although the fine-sized and uniformly distributed grains in the WM can improve its hydrogen embrittlement resistance to a certain extent, the microstructure of WM, which contains larger M/A constituents, MnS, and Al-rich inclusions, significantly impacts embrittlement sensitivity. In contrast, a large number of nanoscale Nb, V, and Ti carbon-nitride composite second phase precipitations in the BM acts as an effective irreversible hydrogen trap, which can significantly improve its hydrogen embrittlement sensitivity.

## Figures and Tables

**Table 1:** Chemical composition of experimental steel (wt. %).

	C	Si	Mn	P	S	Cr	Ni	Cu	Nb	V	Ti	Mo	Al
BM	0.060	0.230	1.400	0.009	0.002	0.186	0.135	0.018	0.053	0.024	0.020	0.100	0.036
WM	0.074	0.250	1.483	0.008	0.005	0.130	1.130	0.026	0.015	0.003	0.037	0.013	0.014

**Table 2:** Ultimate tensile strength, reduction of area, and elongation of BM and WM.

Sampling Position	Environment	UTS (MPa)	RA (%)	EL (%)
BM	10 MPa N_2_	541.9	83.3	27.0
	10 MPa H_2_	549.1	48.4	17.0
Loss rate (%)		−1.3	41.9	37.0
WM	10 MPa N_2_	585.3	81.6	15.6
	10 MPa H_2_	577.1	40.4	9.5
Loss rate (%)		1.4	50.5	39.1

**Table 3:** Number of failure cycles and loss rate of X70 pipeline steel BM and WM specimens.

Sampling Position	Base Metal		WZ Metal	
Environment	10 MPa N_2_	10 MPa H_2_	10 MPa N_2_	10 MPa H_2_
Number of cycles	16,795	13,654	15,096	6437
Loss rate (%)	18.70		57.36	

## Data Availability

The original contributions presented in the study are included in the article, further inquiries can be directed to the corresponding author.
